# Chemicals Policy Gap: Toward Stronger Regulation in the United States

**DOI:** 10.1289/ehp.117-a358b

**Published:** 2009-08

**Affiliations:** **Valerie J. Brown**, based in Oregon, has written for *EHP* since 1996. In 2009 she won a Society of Environmental Journalists’ Outstanding Explanatory Reporting award for her writing on epigenetics

Chemicals policy in the United States is in need of profound change for both environmental health and economic reasons, according to a review of how the U.S. chemical industry currently is regulated **[*EHP*** **117:1202–1209; Wilson and Schwarzman]**. The 1976 Toxic Substances Control Act (TSCA), which provides the chief legal authority for regulating industrial chemicals in the United States, is antiquated and ineffective, according to the authors.

Reform of TSCA is especially urgent in view of the European Union’s passage in 2006 of the Registration, Evaluation, Authorisation and Restriction of Chemicals (REACH) legislation. REACH’s more stringent and transparent rules for regulating industrial chemicals could put the United States at risk of becoming a market for hazardous chemicals that become banned in Europe. Without a parallel transformation in U.S. chemicals policy and a strong commitment to green chemistry, the United States could face growing health and environmental problems and will have difficulty meeting the challenges of environmental and economic sustainability, the authors write.

TSCA currently requires chemical producers to disclose little toxicologic or other test data, creating a data gap that prevents the public and downstream users of chemicals from making informed purchasing decisions. The U.S. Environmental Protection Agency bears the burden of proving that a chemical should be regulated (as opposed to the manufacturer proving that the chemical need not be regulated), and a lack of governmental tools to evaluate and mitigate chemical hazards has produced a safety gap. In concert with the first two gaps, the lack of investment in green chemistry research and development has produced a technology gap, with the United States at risk of falling behind the European Union and other industrialized nations in this area.

The authors argue that the three gaps have produced a chemicals market that values function, price, and performance over safety while externalizing the costs of chemically related health and environmental damage to the public. These market and regulatory conditions also pose a key barrier to the scientific and commercial success of green chemistry in the United States and could hinder the U.S. chemical industry’s global competitiveness as green chemistry technologies accelerate under REACH.

Global chemical production is expected to double in the next 24 years, according to the United Nations and other sources, and the Environmental Protection Agency has estimated the United States will need 217,000 new hazardous waste sites in the next 20 years. Concluding that “the vast potential of green chemistry remains untapped,” the authors call for “a chemicals policy that departs markedly from the federal policies of the last 30 years, of which TSCA is emblematic.” Taking advantage of this opportunity, they write, would propel the United States toward new chemistries that are safer for occupational, environmental, and public health—the cornerstone of a truly sustainable society.

